# Factors associated with persistent postsurgical pain after total knee or hip joint replacement: a systematic review and meta-analysis

**DOI:** 10.1097/PR9.0000000000001052

**Published:** 2023-01-10

**Authors:** Arunangshu Ghoshal, Shivam Bhanvadia, Som Singh, Lauren Yaeger, Simon Haroutounian

**Affiliations:** a Tata Memorial Hospital, Homi Bhaba National Institute, Mumbai, India; b St. Louis University School of Medicine, St. Louis, MO, USA; c University of Missouri Kansas City School of Medicine, Kansas City, MO, USA; d Becker Medical Library, Washington University School of Medicine, St. Louis, MO, USA; e Department of Anesthesiology and Washington University Pain Center, Washington University School of Medicine, St. Louis, MO, USA

**Keywords:** Persistent postsurgical pain, Chronic postsurgical pain, Knee replacement, Hip replacement, Systematic review, Meta-analysis

## Abstract

Studies have identified demographic, clinical, psychosocial, and perioperative variables associated with persistent pain after a variety of surgeries. This study aimed to perform a systematic review and meta-analysis of factors associated with persistent pain after total knee replacement (TKR) and total hip replacement (THR) surgeries. To meet the inclusion criteria, studies were required to assess variables before or at the time of surgery, include a persistent postsurgical pain (PPSP) outcome measure at least 2 months after a TKR or THR surgery, and include a statistical analysis of the effect of the risk factor(s) on the outcome measure. Outcomes from studies implementing univariate and multivariable statistical models were analyzed separately. Where possible, data from univariate analyses on the same factors were combined in a meta-analysis. Eighty-one studies involving 171,354 patients were included in the review. Because of the heterogeneity of assessment methods, only 44% of the studies allowed meaningful meta-analysis. In meta-analyses, state anxiety (but not trait anxiety) scores and higher depression scores on the Beck Depression Inventory were associated with an increased risk of PPSP after TKR. In the qualitative summary of multivariable analyses, higher preoperative pain scores were associated with PPSP after TKR or THR. This review systematically assessed factors associated with an increased risk of PPSP after TKR and THR and highlights current knowledge gaps that can be addressed by future research.

## 1. Introduction

Persistent postsurgical pain (PPSP) is a common sequel of surgical procedures. The prevalence varies for different surgical procedures and in different studies, ranging from a low of 3% to a high of 81%,^90^ with a reported incidence of about 20% after bone and joint surgeries.^39^ PPSP substantially impairs quality of life, has negative economic consequences,^35^ and is a potentially preventable condition.^106^ Patients who undergo elective procedures such as total hip or knee replacement, typically expect alleviation of the pain associated with the damaged joint. Although these procedures have a high likelihood of success in relieving pain and restoring function, PPSP is a major factor that negatively affects patient satisfaction with joint replacement surgery outcomes.^72^ Therefore, understanding factors predisposing patients to an increased risk of persistent pain is important in attempts to prevent PPSP or minimize its occurrence. The identification of patients at high risk for PPSP will allow allocating perioperative resources for PPSP risk mitigation and may signal a need for enhanced postoperative follow-up and early referral to multidisciplinary evaluation. Several studies been have published on perioperative risk factors associated with PPSP,^120^ but it is yet unclear which factors show a consistent association with PPSP.^47,67^

Several systematic reviews have been published on psychosocial factors in general^43^ or anxiety and catastrophizing specifically^107^ as risk factors for PPSP, and individual studies have reported on factors associated with orthopedic surgeries such as total hip replacement (THR)^27^ and total knee replacement (TKR).^109^ An up-to-date synthesis of the existing literature can help improve perioperative planning and care, as well as guide future study designs aimed at better understanding and possible prevention of PPSP.

The objective of the current study was to perform a systematic review and meta-analysis on the reported factors associated with PPSP after total hip joint and knee joint replacement.

## 2. Methods

The protocol for this systematic review has been preregistered on PROSPERO (CRD42020152146).

### 2.1. Inclusion and exclusion criteria for studies

The studies were included if they reported PPSP occurrence after a hip joint or knee joint replacement. We included both prospective and retrospective studies that addressed PPSP as pain at the surgical or related site, present at least 2 months postoperatively. The minimum 2-month cutoff was selected as several definitions of PPSP in the past decade have been proposed,^60^ with most suggesting pain duration for more than 2 or 3 months after surgery. Exclusion criteria included (1) fewer than 10 participants per arm, (2) follow-up shorter than 2 months after surgery, (3) participants younger than 18 years, (4) studies on patients with established PPSP that did not report the occurrence of persistent pain after surgery, (5) studies not reporting incidence or prevalence rate of PPSP in the cohort, and (6) abstracts.

A systematic literature search was performed in the following databases: Ovid MEDLINE, Embase, Scopus, Cochrane Central Register of Control Trials, Cumulative Index to Nursing and Allied Health Literature and PsychINFO. The full keyword search strategy can be found in Appendix 1 (available at http://links.lww.com/PR9/A184). The search was initially performed on September 17, 2019, with an update on October 14, 2020 (Appendix 1A, http://links.lww.com/PR9/A184), and subsequently expanded on July 9, 2021 (Appendix 1B, http://links.lww.com/PR9/A184), and updated on February 6, 2022 (Appendix 1C, http://links.lww.com/PR9/A184). The duplicates in the resulting abstracts were removed using EndNote.^12^

The abstracts of the resulting list of articles were screened by 2 authors for eligibility. If the studies were deemed eligible based on abstracts, then the full texts of those articles were retrieved and reviewed independently. The data from eligible articles were extracted into REDCap (Research Electronic Data Capture) and Excel spreadsheets. The data were extracted by 2 authors (A.G. and S.B./S.S.) and compared for inconsistencies. Wherever disagreements arose, a third author (S.H.) was consulted to reach the final decision. Risk of bias (ROB) assessments and data extraction were performed by the 2 reviewers (A.G. and S.B./S.S.) and compared for accuracy.

### 2.2. Risk of bias assessment

Most validated tools for assessing the ROB in systematic reviews^42^ refer to assessing the methodology of randomized controlled trials. Because this study aimed to analyze factors associated with PPSP (from both prospective and retrospective studies), an alternative method for the ROB systematic assessment was applied based on the methodology used in other systematic reviews focusing on the analysis of predicting factors.^53,56,116^ Potential sources of bias were evaluated and addressed whether (1) the sample adequately represented the population of interest, (2) the data represented the sample, and (3) the outcome of interest was adequately measured to limit potential bias (Appendix 2, available at http://links.lww.com/PR9/A184). Other potential serious sources of bias were documented—eg, selective reporting of outcomes, use of nonvalidated scales, or lack or inappropriateness of sample size calculation.

### 2.3. Protocol for data extraction

To address the different study designs, we developed guiding principles for data extraction across all studies (Appendix 3, available at http://links.lww.com/PR9/A184). In brief, all studies which addressed PPSP at or more than 2 months were included, and the follow-up timeframe at or closest to 6 months was obtained in case of multiple follow-ups. Although the cutoff for postsurgical duration of pain has most commonly been set at 2 or 3 months, some patients have a longer rehabilitation period after total knee or hip joint replacement surgeries.^38,55^ Thus, to remove the confounder, we decided to set the primary outcome timeframe at around 6 months after surgery in studies with multiple follow-ups. If PPSP was categorized based on a severity scale (numerical rating scale [NRS] or Likert scale), the data were dichotomized to a “no pain to mild pain” group vs a “moderate-severe pain” group, to allow 2 × 2 table formation. In addition, to allow systematic analysis of data, some factors were combined, eg, epidural and spinal anesthesia were merged under neuraxial anesthesia.

### 2.4. Data synthesis and analysis

The data analyses were performed using RStudio version 1.4.1103.^93^ For binary factors, 2 × 2 tables were created, and risk ratios were calculated to be used in generating forest plots. For continuous variables (eg, age and body mass index [BMI]), weighted mean differences were used. Only raw univariate data were used for generating forest plots. Values for weighted mean differences were presented as means with standard deviation (SD). Risk Ratio (RR) or odds ratio was presented as risk or odds, with 95% confidence interval (CI). Meta-regression of multivariable data was not possible because each study accounted for different risk factors. Data on risk factors from multivariable analyses were aggregated in a tabulated form to help make qualitative inferences about common contributing factors.

## 3. Results

**Figure 1.**
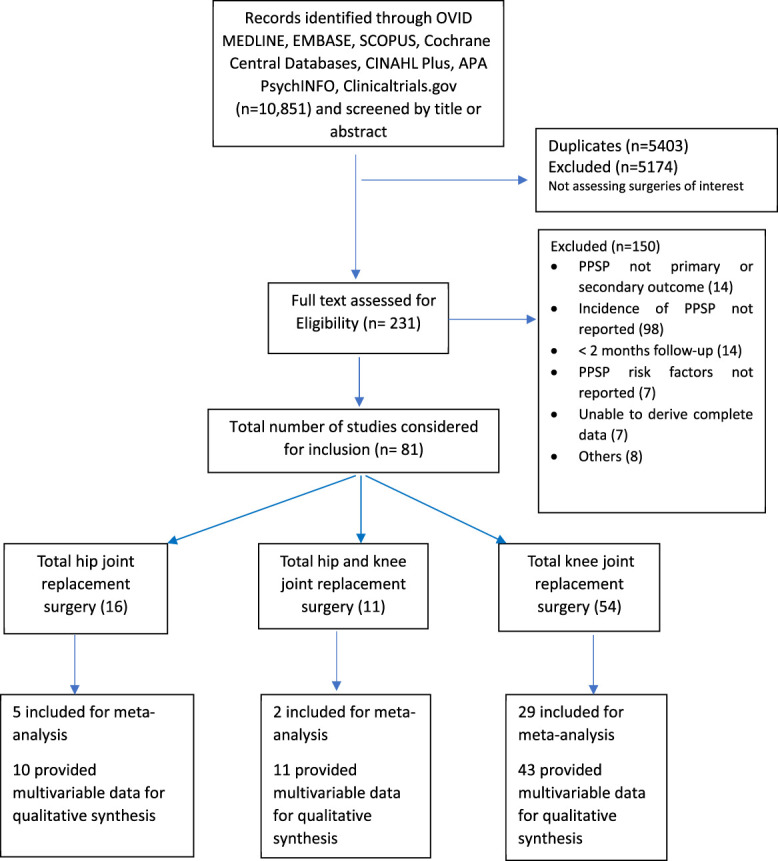
Preferred Reporting Items for Systematic Reviews and Meta-Analyses flow chart.

The systematic literature search resulted in a total of 10,851 potential articles. After removing 5,403 duplicate records, a total of 5,448 unique citations were added to the project library. All abstracts of identified studies were reviewed for inclusion criteria, resulting in 231 full-text articles that were considered for full review (see Appendix 1 for full details about literature search strategy and results, available at http://links.lww.com/PR9/A184). These articles were independently evaluated for inclusion by 2 reviewers (AG and SB/SS), and 81 were included in the final analysis (Fig. 1).

The studies analyzed included total hip and/or knee joint replacement surgeries. The overall study characteristics are outlined in Table 1. The minimum cutoff for determining PPSP in the included studies was 3 months after surgery.^98^ The total number of patients across all total hip and/or knee joint replacement surgery studies (n = 81) was 171,354 with a mean (SD) sample size of 2,115 (14,226), 28 (34.6%) studies involving less than 100 participants. The mean PPSP rate was 16.4%. Studies investigating PPSP after both total hip and knee joint replacement surgery (n = 11) included 10,399 participants and reported a mean PPSP incidence of 26.7%. Studies after total hip joint replacement surgery alone (n = 16) included 9,086 participants and reported a mean PPSP incidence of 16.5%, and those after total knee joint replacement surgery alone (n = 54) included 151,869 participants and reported a mean PPSP incidence of 15.6%. Overall, 69 of the studies were prospective and 12 were retrospective. A total of 36 studies have provided univariate data to be included in meta-analysis, and 64 studies have reported risk factors in multivariable analyses, from which data were used for qualitative summary (Fig. 1). The studies overall had medium to low ROB (Table 2). The main category to have significant ROB was a “follow-up rate >75% at the first follow-up” (with 14 studies (17%) with high ROB), incomplete characterization of study dropouts (with 65 studies [80%] having unclear or high ROB).Thirty five of the 81 included studies (43%) had high or unclear bias in one of the categories of pain outcome measurement—either in PPSP definition, its method of assessment or classification, or reporting—and 62 of 81 (76.5%) studies reported sources of funding. Although there is not a good way to assess publication bias related to risk prognostic studies,^105^ funnel plots to assess asymmetry among most commonly reported risk factors did not suggest important publication bias (Appendix 4, available at http://links.lww.com/PR9/A184).

**Table 1:** Main characteristics of included studies (n = 81).

SN	Author-date	Surgery	Type of study	Total number of participants	Occurrence of PPSP percentage (number)	Follow-up duration*
1	Albayrak 2016	TKR	Prospective	274	66.7% (183)	22 mo
2	Aso 2021	TKR	Prospective	194	10.3% (20)	6 mo
3	Attal 2014	TKR	Prospective	81	39.5% (32)	6 mo
4	Aveline 2014	TKR	Prospective	75	17.4% (13)	12 mo
5	Baker 2007	TKR	Cross-sectional mail/phone survey	8010	19.8% (1583)	24 mo
6	Bischoff-Ferrari 2004	THR	Retrospective	897	42% (377)	36 mo
7	Bossmann 2017	TKR	Prospective	47	20% (9)	6 mo
8	Bourne 1994	THR	Prospective	94	27.6% (26)	60 mo
9	Brander 2003	TKR	Prospective	116	18.4% (21)	12 mo
10	Briggs 1995	TKR	Prospective	65	6% (4)	24 mo
11	Bugada 2017	TKR	Prospective	614	21.6% (133)	6 mo
12	Buvanendran 2010	TKR	Randomized controlled study	228	5.2% (placebo arm: 6 of 115)	6 mo
13	Buvanendran 2019	TKR	Prospective	245	14% (34)	6 mo
14	Buvanendran 2020	TKR	Prospective	50	34% (17)	3 mo
15	Chen 2021	TKR	Prospective	220	13.6% (30)	6 mo
16	Clarke 2009	THR	Randomized controlled study	126	(G1: placebo/placebo 26.3% (10); G2: GPN/placebo 31.6% (12); G3: placebo/GPN 23.7% (9)).	6 mo
17	Clarke 2010	THR	Prospective	82	37.5% (31)	6 mo
18	Dong 2019	TKR	Randomized controlled study	122	36.06% (44)	3 mo
19	Dowsey 2015	TKR	Retrospective	689	22% (151)	12 mo–60 mo
20	Dumenci 2019	TKR	Randomized clinical trial	384	18% (69)	12 mo
21	Dürsteler 2021	TKR	Prospective	146	31.5% (46)	3 mo
22	Erlenwein 2017	THR	Prospective	104	17.3% (18)	6 mo
23	Fletcher 2015	THR and TKR	Prospective	145	27.6% (40)	12 mo
24	Forsythe 2008	TKR	Prospective	48	77.1% (37)	24 mo
25	George 2021	THR and TKR	Retrospective	2411 (1146 THR, 1265 TKR)	THR 33.6% (385)	6 mo
					TKR 38.9% (492)	
26	Giordano 2020	TKR	Prospective	136	16.2% (22)	12 mo
27	Grosu 2016	TKR	Prospective	114	10% (11)	6 mo
28	Guimaraes-Pereira 2016	THR and TKR	Prospective	19	50% (9)	3 mo
29	Gungor 2019	TKR	Retrospective	578	31.1% (180)	3 mo
30	Kornilov 2017	TKR	Prospective	79	22.8% (18)	12 mo
31	Kuchálik 2017	THR	Retrospective	72	Group ITM 6% (2)	6 mo
					Group LIA 0% (0)	
32	Kuchálik 2017	THR	Randomized controlled study	56 (group LIA (n = 29), group FNB (n = 27))	Group LIA 0% (0)	6 mo
					Group FNB 0% (0)	
33	Kurien 2018	TKR	Prospective	50	30.4% (14)	6 mo
34	Lavand'homme 2014	TKR	Prospective	112	11% (12)	3 mo
35	Lee 2019	TKR	Randomized controlled study	24	25% (6)	6 mo
36	Lindberg 2021	TKR	Prospective	202	30.2% (61)	3 mo
37	Liu 2012	THR and TKR	Cross-sectional mail/phone survey	1030	45.9% (473) (THR:38% (178)	12 mo
					TKR:62% (295))	
38	Lu 2021	THR	Prospective	736	27.2% (200)	6 mo
39	Martinez 2007	TKR	Prospective	20	20% (4)	4 mo
40	Masselin-Dubois 2013	TKR	Prospective	89	50.6% (45)	3 mo
41	Nazal 2019	THR	Retrospective	10	2% (2)	24 mo
42	Neuprez 2020	THR and TKR	Prospective	626 (346 THR, 280 TKR)	THR 8.99% (31)	60 mo
					TKR 3.23% (9)	
43	Nikolajsen 2006	THR	Cross-sectional mail/phone survey	1048	28.1% (294)	12–18 mo
44	Noiseux 2014	TKR	Prospective	215	4.6% (10)	6 mo
45	Oh 2019	TKR	Retrospective	924	16% (148)	12 mo
46	Pagé 2015	TKR	Prospective	108	8.3% (9)	12 mo
47	Pagé 2016	THR	Prospective	111	21.6% (24)	6 mo
48	Peng 2014	TKR	Randomized controlled study	212 (group CFNB [n = 109] group PCIA [n = 103])	Group CFNB 33% (36)	6 mo
					Group PCIA 52.4% (54)	
49	Pereira 2016	THR and TKR	Prospective	43 (TKR 22, THR 21)	TKR 68.2% (15)	6 mo
					THR 23.8% (5)	
50	Petersen 2015	TKR	Cross-sectional mail/phone survey	305 (215 primary surgery patients, 90 revision surgery patients)	19% (41) primary surgery patients	36 mo
					47% (42) revision surgery patients	
51	Petersen 2015	TKR	Prospective	78	22% (17)	12 mo
52	Petersen 2017	TKR	Prospective	130	19% (25)	12 mo
53	Petersen 2020	TKR	Prospective	26	35% (9)	12 mo
54	Pinedo-Villanueva 2018	TKR	Retrospective	128145	15% (19222)	6 mo
55	Pinto 2013	THR and TKR	Prospective	92 (THR 48, TKR 44)	41.3% (38)	4–6 mo
					THR (13) TKR (25)	
56	Puolakka 2010	TKR	Cross-sectional mail/phone survey	562	35% (197)	4–22 mo
57	Rao 2020	TKR	Randomized controlled study	40	47.5% (19)	3–6 mo
58	Remérand 2009	THR	Randomized controlled study	142 (70 placebo, 72 ketamine)	14.8% (21)	6 mo
					15 placebo, 6 ketamine	
59	Rice 2018	TKR	Prospective	286	21% (60)	6 mo
60	Sakellariou 2015	TKR	Cross-sectional mail/phone survey	272	39.34% (107)	12–16 mo
61	Sanders 2009	TKR	Randomized controlled study	56 (baclofen treatment group n = 27, control group n = 29)	27 (baclofen treatment group n = 8, control group n = 19)	3 mo
62	Sayers 2016	THR and TKR	Randomized controlled study	560 (283 THR, 277 TKR)	5% (14) THR	12 mo
					12% (33) TKR	
63	Sen 2020	TKR	Retrospective	182	48.4% (88)	6.84 ± 4.10 mo
64	Sideris 2021	TKR	Prospective	162	9.3% (15)	6 mo
65	Singh 2010	THR	Cross-sectional mail/phone survey	5390	8.1% (435)	24 mo
66	Skrejborg 2019	TKR	Cross-sectional mail/phone survey	604	18% (107)	60 mo
67	Sugiyama 2018	TKR	Retrospective	298	33% (97)	6 mo
68	Thomazeau 2016	TKR	Prospective	104	28.8% (74)	6 mo
69	Vaegter 2017	TKR	Prospective	14	28.6% (4)	6 mo
70	Valdes 2012	THR and TKR	Prospective	1788 (THR 928, TKR 860)	THR 17.0% (158)	38 mo
					TKR 25.3% (217)	
71	Vila 2020	TKR	Prospective	112	41.96% (47)	6 mo
72	Von Dincklage 2017	THR	Prospective	105	13.3% (14)	6 mo
73	Wang 2014	THR	Randomized controlled study	51 (spinal saline 28, spinal ketorolac 23)	15% (8)	6 mo
74	W-Dahl 2014	TKR	Retrospective	2123	7.8% (165)	12 mo
75	Wylde 2009	THR and TKR	Cross-sectional mail/phone survey	2391 (1112 THR, 613 TKR)	THR 13% (144)	60–96 mo
					TKR 26% (159)	
76	Wylde 2011	THR and TKR	Cross-sectional mail/phone survey	1294 (662 THR, 632 TKR)	THR 27% (179)	24–48 mo
					TKR 44% (278)	
77	Wylde 2013	TKR	Prospective	51	29% (15)	13 mo
78	Wylde 2020	TKR	Retrospective	3058	11.87% (363)	3 mo
79	Yang 2020	TKR	Randomized controlled study	96	27.08% (26)	3 mo
80	Yao 2019	TKR	Retrospective	694	9.94% (69)	6 mo
81	Zachodnik 2021	THR	Prospective	62	24.19% (15)	12 mo

* Follow-up time points used for data analysis.

CFNB, continuous femoral nerve block, PCIA, patient-controlled intravenous analgesia; GPN, gabapentin; TKR, total knee replacement; THR, total hip replacement; ITM, intrathecal morphine; LIA, local infiltration analgesia; FNB, femoral nerve block.

**Table 2:** Risk of bias assessment of the included studies.

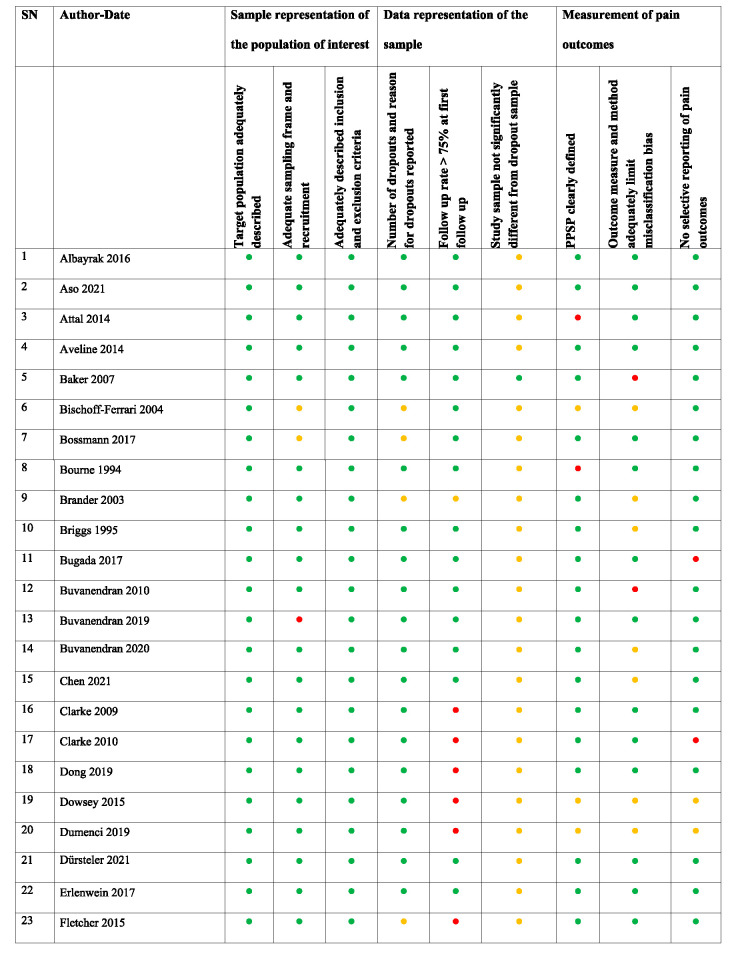
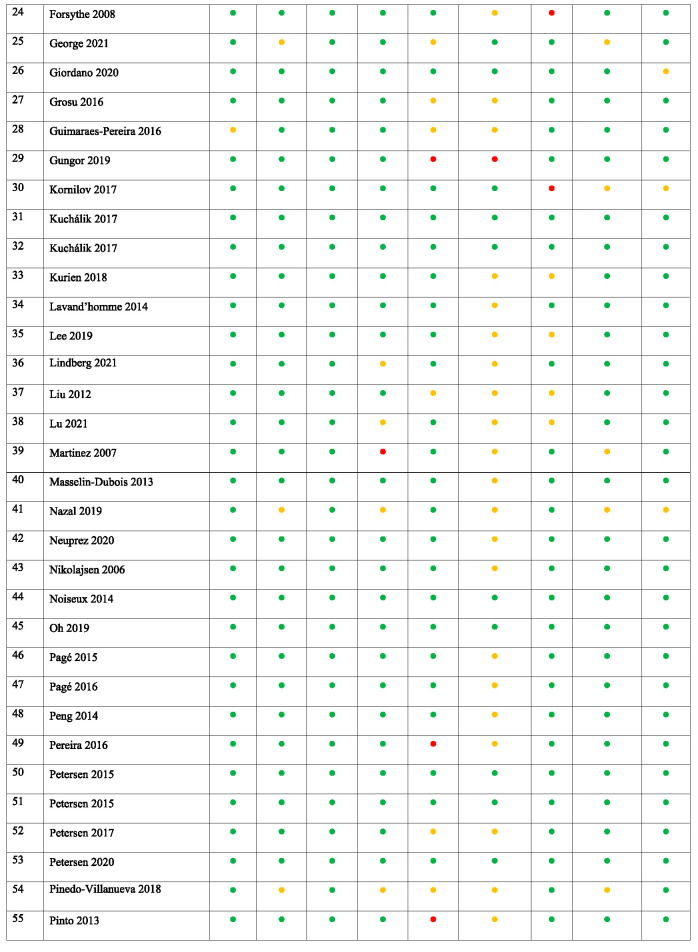
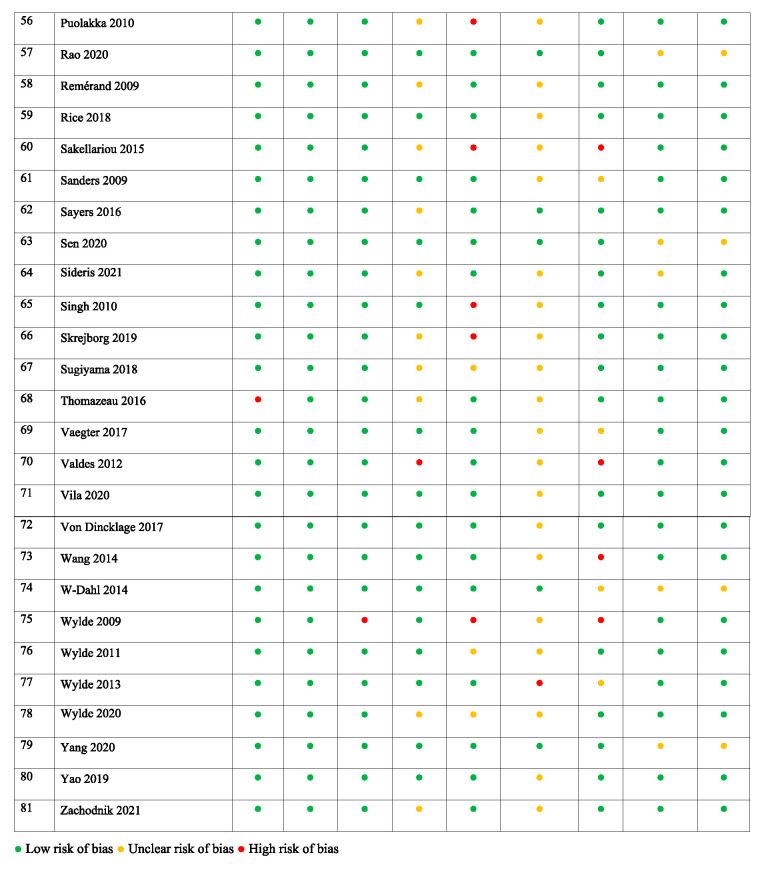

### 3.1. Results of meta-analysis

#### 3.1.1. Univariate comparisons

The demographic factors with data from multiple studies that allowed testing their association with an increased risk of PPSP were age, the proportion of women in the total study population, and BMI, whereas clinical factors included duration of surgery (in minutes), preoperative factors such as presence of anxiety and depression, knee or hip joint pain, or pain elsewhere in the body.

**Figure 2.**
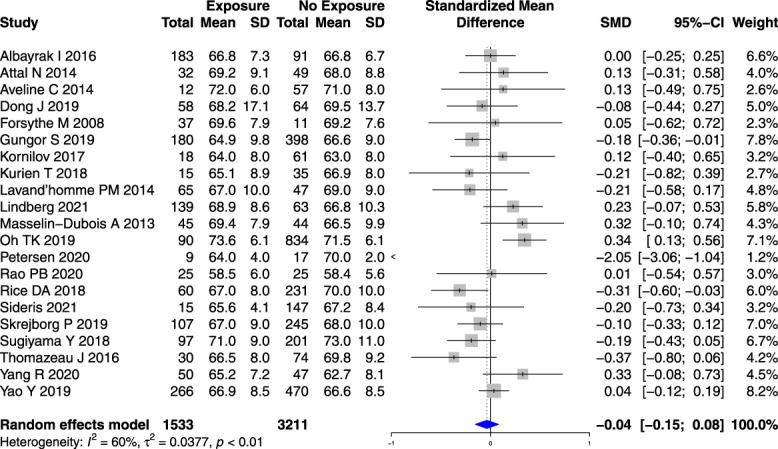
Forest plot for the association between age and PPSP in total knee joint replacement surgeries analyzed together. *Higher/positive SMD represents the difference in age (in years) between patients who developed PPSP vs those who did not, the horizontal box and whisker plots are the group SMDs ±95% confidence intervals, and the blue diamond is the combined SMD of groups.

**Figure 3.**
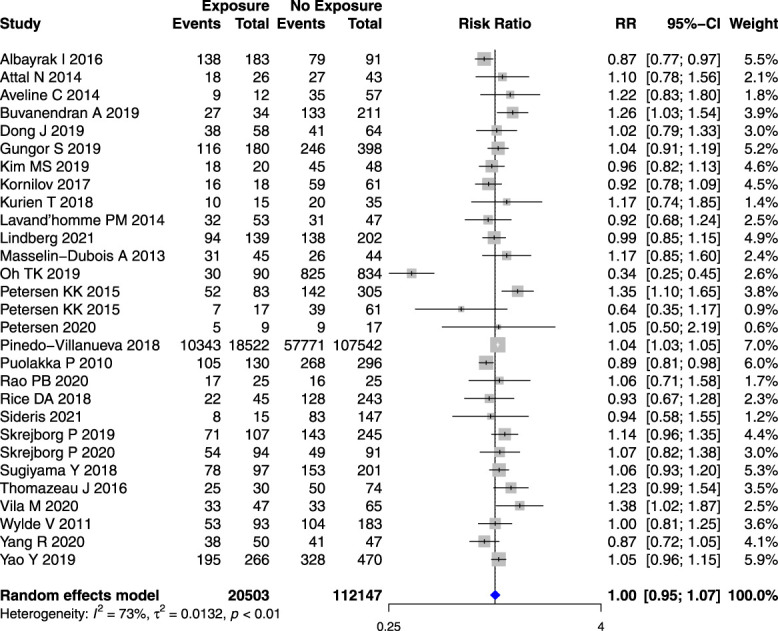
Forest plot for the association between the proportion of women in the study population and PPSP in total knee joint replacement surgeries. *Risk ratio (RR) >1 represents higher, RR <1 represents lesser, while RR = 1 represents a similar occurrence of PPSP in women compared with the study population, the horizontal box and whisker plots are the group RRs ±95% confidence intervals, and the blue diamond is the combined RR of groups.

**Figure 4.**
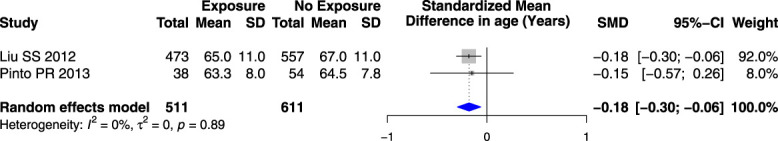
Forest plot for the association between age and PPSP in total knee and hip joint replacement surgeries analyzed together. *Higher/positive SMD represents the difference in age (in years) between patients who developed PPSP vs those who did not, the horizontal box and whisker plots are the group SMDs ±95% confidence intervals, and the blue diamond is the combined SMD of groups.

Age (Fig. 2) and sex (Fig. 3) were not associated with an increased risk of PPSP after TKR or THR (Appendix 5 and Appendix 6 respectively, available at http://links.lww.com/PR9/A184) studies when analyzed separately. However, younger age (SMD −0.18 years [95% CI −0.30 to −0.06]) (Fig. 4) and female sex (RR 1.13, 95% CI 1.02–1.24) (Appendix 7, available at http://links.lww.com/PR9/A184) were associated with an increased risk in a small subset of studies reporting on the combined knee and hip replacement surgeries (n = 2).

**Figure 5.**
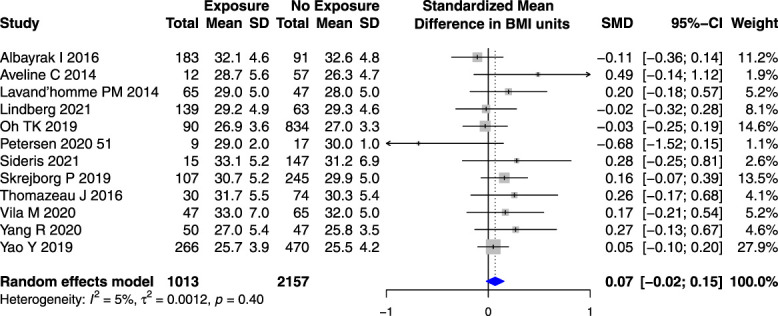
Forest plot for the association between body mass index (BMI) in kg/m^2^ and PPSP in total knee joint replacement surgeries analyzed together. *Higher/positive SMD represents the difference in BMI units between patients who developed PPSP vs those who did not, the horizontal box and whisker plots are the group SMDs ±95% confidence intervals, and the blue diamond is the combined SMD of groups.

Body mass index was not associated with a risk of PPSP in TKR studies (Fig. 5), but higher BMI was associated with higher PPSP occurrence in the subset of studies reporting both hip and knee replacement (SMD 0.15 kg/m^2^ [95% CI 0.03–0.26]) (Appendix 8, available at http://links.lww.com/PR9/A184).

**Figure 6.**
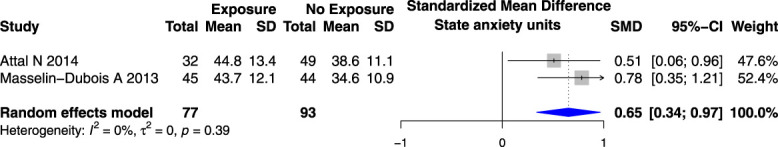
Forest plot for the association between state anxiety and PPSP in total knee joint replacement surgeries analyzed together. *Higher/positive SMD represents the difference in the state anxiety score between patients who developed PPSP vs those who did not, the horizontal box and whisker plots are the group SMDs ±95% confidence intervals, and the blue diamond is the combined SMD of groups.

**Figure 7.**
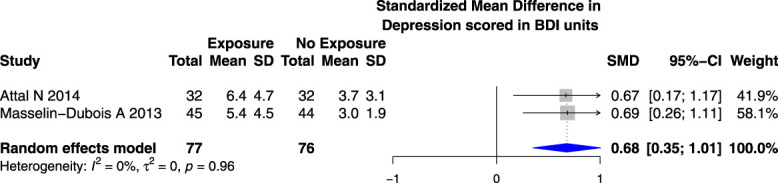
Forest plot for the association between depression scored in the Beck Depression Inventory (0–39) and PPSP in total knee joint replacement surgeries was analyzed together. *Higher/positive SMD represents the difference in depression scored in the Beck Depression Inventory (score range 0–39) units between patients who developed PPSP vs those who did not, the horizontal box and whisker plots are the group SMDs ±95% confidence intervals, and the blue diamond is the combined SMD of groups.

Higher state anxiety (Fig. 6) (but not trait anxiety) (Appendix 9, available at http://links.lww.com/PR9/A184) scores (SMD 0.65 [95% CI 0.34–0.97]) (measured on The Spielberger State-Trait Anxiety Inventory) and higher depression scores on the Beck Depression Inventory (SMD 0.68 [95% CI 0.35–1.01]) (Fig. 7) were associated with an increased risk of PPSP in studies reporting PPSP after total knee joint replacement surgery, but not for depression (Appendix 10, available at http://links.lww.com/PR9/A184) or anxiety (Appendix 11, available at http://links.lww.com/PR9/A184) scored in the Hospital Anxiety and Depression Scale.

**Figure 8.**
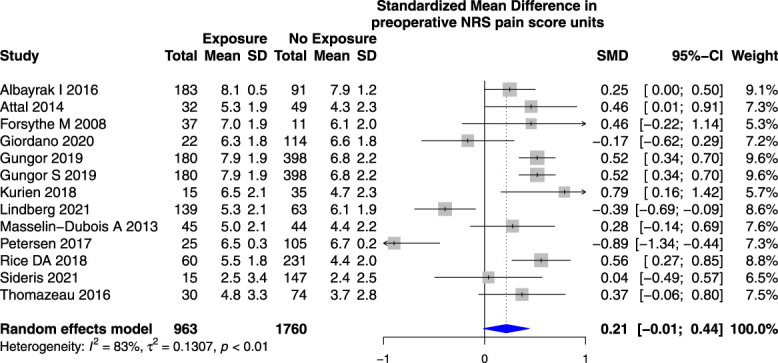
Forest plot for the association between preoperative numerical rating scale pain score (0–10) and PPSP in total knee joint replacement surgeries analyzed together. *Higher/positive SMD represents the difference in preoperative numerical rating scale pain score (0–10) units between patients who developed PPSP vs those who did not, the horizontal box and whisker plots are the group SMDs ±95% confidence intervals, and the blue diamond is the combined SMD of groups.

Higher preoperative NRS pain scores (0–10) were not associated with an increased risk of PPSP in studies reporting PPSP after total knee joint replacement surgery (SMD 0.21 [95% CI −0.01 to 0.44)] (Fig. 8) or total knee and hip joint replacement surgeries analyzed together (Appendix 12, available at http://links.lww.com/PR9/A184).

Other associations tested were duration of surgery (mins) (Appendix 13, available at http://links.lww.com/PR9/A184) and pre-existing pain at other sites of the body (Appendix 14, available at http://links.lww.com/PR9/A184), but these did not reach statistical significance.

### 3.2. Qualitative summary of multivariable analyses

Table 3 presents a qualitative summary of risk factors that were reported in multivariable analyses in the included studies. Factors are reported as either having a significant association with an increased occurrence of PPSP (red) or no association (yellow). The variables for which the risk factors were adjusted for differ among the studies.

**Table 3:** Qualitative summary of risk factors for PPSP reported in multivariable analyses in included studies.

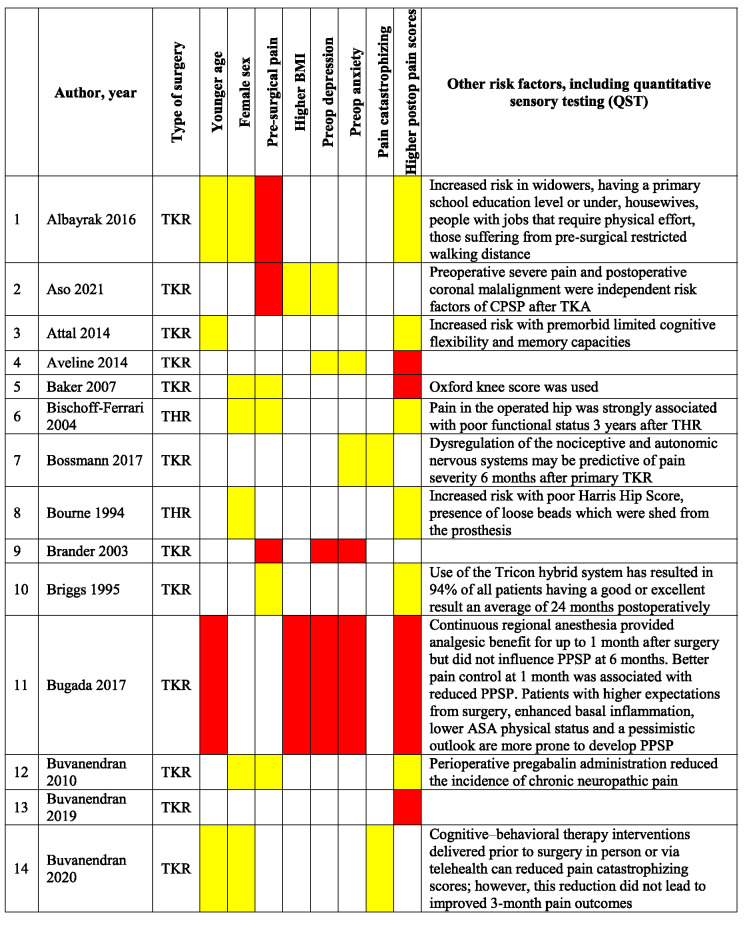
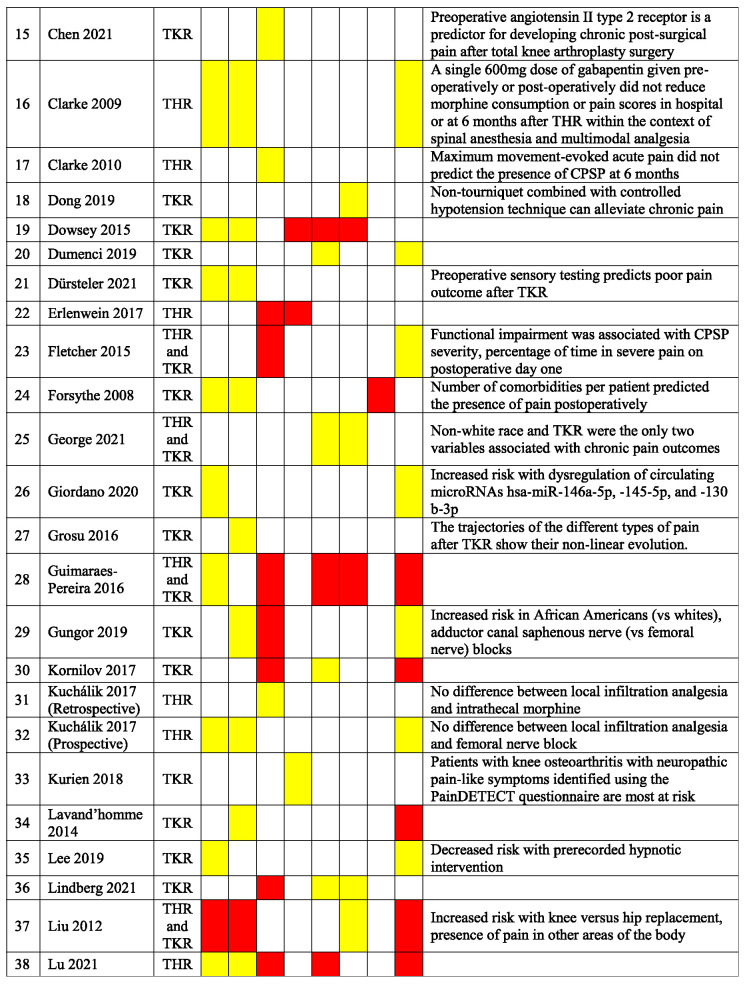
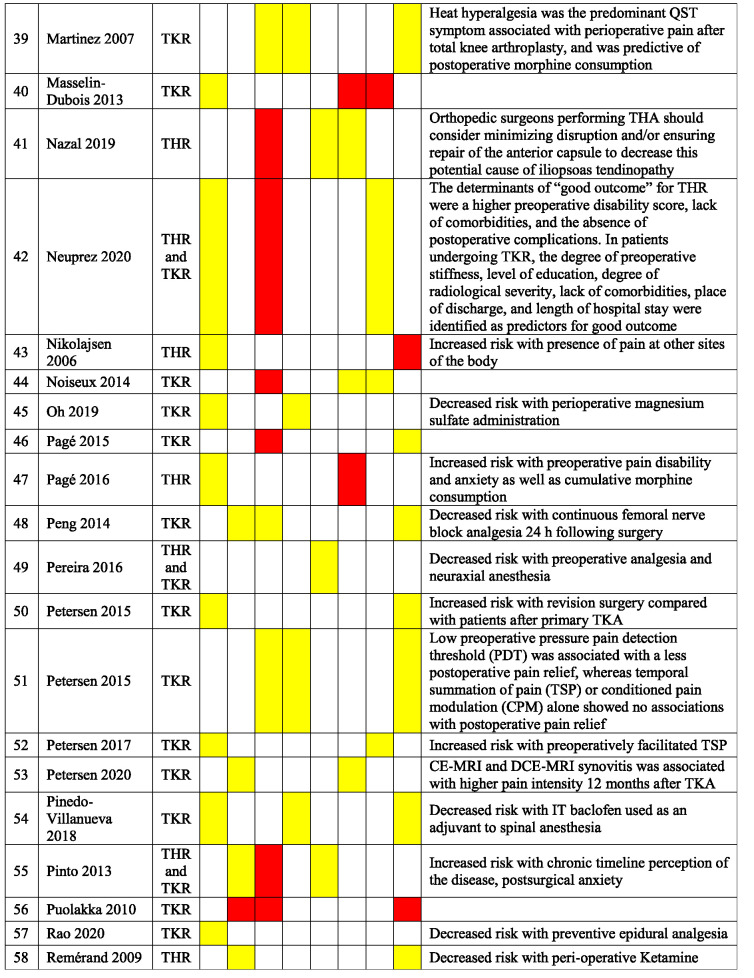
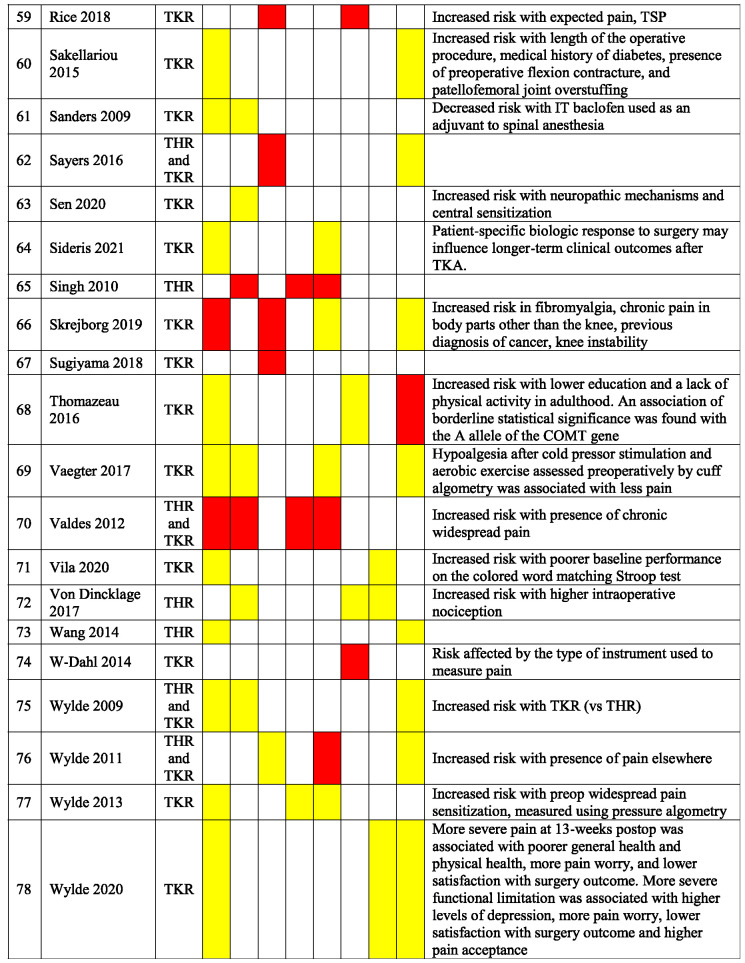
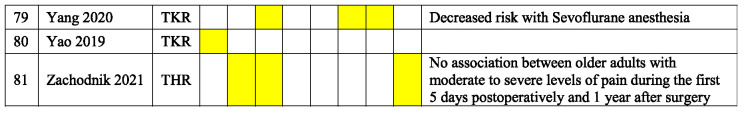

Results are color coded for the type of associations.

Red, increased odds of PPSP; Green, decreased odds of PPSP; Yellow, no association with PPSP; White, not reported.

### 3.3. Demographic factors

In multivariable analysis, an increased risk of PPSP was associated with younger age in 4^16,57,100,111^ of 36 studies, female sex in 4^57,85,99,111^ of 29 studies, and with BMI in 4^15,27,99,111^ of 12 studies that tested these variables as independent risk factors.

### 3.4. Preoperative factors

Large variability in the incidence of PPSP was found by 2 studies depending on the definition^19^ (any pain within the last 4 weeks: NRS >0 vs current pain at the time of the interview NRS >0 vs moderate-to-severe pain at the time of the interview: NRS >3) and assessment instrument used^76^ (Knee Injury and Osteoarthritis Outcome Score pain subscale vs visual analogue scale) to measure pain. Increased risk of PPSP was associated with higher pre-existing pain intensity in 17^1,13,85,89,97,100,104,27,20,27,28,50,71,74,82,2,58^ of 30 studies and with unresolved postoperative pain in the affected knee or hip joint in 12^4,6,108,15,20,36,41,45,48,61,85,49^ of 41 studies that tested these variables as independent risk factors.

### 3.5. Pre-existing health conditions

Higher PPSP was observed in patients with comorbidities such as hypertension, diabetes, ischemic heart disease, chronic obstructive pulmonary disease, asthma, and neurological illnesses in 7 studies,^3,30,94,100,69,126,25^ and patients with the presence of widespread pain in other parts of the body in 5 studies.^26,70,100,111,123^

### 3.6. Psychological factors

Increased risk of PPSP was associated with pre-existing anxiety in 8^13,15,25,36,65,73,89,114^ of 20 studies, with pre-existing depression in 7^13,15,25,36,99,111,122^ of 20 studies, with preoperative pain catastrophizing in 2^30,65^ of 11 studies, and with poorer baseline performance on cognitive flexibility tests in 2 studies^3,112^ that tested these variables as independent risk factors.

### 3.7. Psychophysical assessments

Low preoperative pressure pain detection threshold was associated with PPSP in 1 study,^77^ whereas temporal summation of pain (TSP) or conditioned pain modulation (CPM) alone showed no associations, but the association with CPM was seen in male subgroup patients.^10^ Increased odds of PPSP were also observed with heat hyperalgesia,^62^ preoperatively facilitated TSP,^45^ expected pain and TSP,^89^ and with preoperative widespread pain sensitization measured using pressure algometry,^125^ whereas hypoalgesia after cold pressor stimulation and aerobic exercise assessed preoperatively by cuff algometry was associated with less PPSP.^110^

### 3.8. Perioperative interventions

There were no sufficient data to combine perioperative interventions in the meta-analyses. Data from 1 or 2 articles were available on each intervention; therefore, only a qualitative summary is provided below.

In the 4 studies which reported on regional anesthesia in TKR, a reduced incidence of PPSP was reported with continuous femoral nerve block analgesia with standardized rehabilitation therapy in 1 study,^76^ whereas analgesic benefit was noticed only for up to 1 month after surgery in 1 study^15^ or even for a shorter immediate postoperative period but not 3 or 6 months postsurgery in the other 2.^51,128^ There was reduced pain at 3 months after TKR with intrathecal baclofen used as an adjuvant to spinal anaesthesia,^95^ with sevoflurane anaesthesia,^127^ and with preventive epidural analgesia.^86^ Perioperative pregabalin administration reduced the incidence of chronic neuropathic pain in a single study.^17^

After THR, a single 600-mg dose of gabapentin given preoperatively or postoperatively neither reduced morphine consumption nor pain scores in the hospital or after 6 months within the context of spinal anesthesia and a robust multimodal analgesia regimen.^21^ There was no difference between local infiltration analgesia and intrathecal morphine or femoral nerve block,^51^ and no effect of a single spinal dose of ketorolac^115^ or perioperative ketamine^88^ on PPSP prevention. There was no effect on PPSP from preoperative analgesia and neuraxial anesthesia in a study analyzing THR and TKR together.^36^

## 4. Discussion

This systematic review and meta-analysis focused on identifying reported factors associated with PPSP risk across a group of hip joint and knee joint replacement surgeries. Overall, 81 studies met the eligibility criteria and were included for further analyses. The studies selected based on inclusion criteria had mostly low or unclear ROB for appropriate representation of the surgical population tested, study design, likelihood of publication bias, and the measurement of pain outcomes. However, based on ROB assessment, important sources of bias and heterogeneity were low follow-up rate and inconsistent characterization of participants who dropped out of studies, as well as inconsistent definition of PPSP, its assessment methods and reporting. The overall mean PPSP rate was 16.4% (16.5% after THR and 15.6% after TKR), which was similar to the previous literature review (9% or more after THR and about 20% after TKR).^59,102,113,121^

### 4.1. Knee joint replacement surgeries

The results of the meta-analyses in knee joint replacement surgeries from 54 studies demonstrated an increased risk of PPSP in individuals with higher state anxiety (but not trait anxiety) scores and higher depression scores (in Beck Depression Inventory). Literature suggests that a bidirectional relationship exists between pain and anxiety or depression, and observations from functional imaging studies suggest that this bidirectional relationship is due in part to shared neural circuitry, particularly related to mechanisms of emotional regulation of pain.^130^

The qualitative summary of multivariable analysis from 43 studies pointed out several possible factors associated with PPSP but with limited data and evidence. Presurgical pain intensity was the only factor where more studies reported an independent risk with PPSP compared with studies that did not find such an association. Although factors such as younger age, female sex, higher BMI, high catastrophizing, more severe acute postoperative pain, pre-existing depression, or anxiety were independently associated with PPSP in some studies, there were generally more studies that did not find such independent association. Certain factors were demonstrated in single studies to increase the risk of PPSP. Among these were being a widower; being a housewife; not having higher education; working at a job that requires physical effort;^1^ presurgical restricted walking distance; pre-existing knee pain; neuropathic pain;^81^ pre-existing chronic pain states;^119^ poorer cognitive flexibility; comorbidities such as diabetes mellitus, cancer, and fibromyalgia;^33^ revision surgery compared with primary TKA;^79^ mechanical complication of prosthesis;^48^ and painful postoperative period.^34^ Given the paucity of data, future research will be needed to test these associations further.

Interventions such as preoperative exercise and education were suggested to have low-quality to moderate-quality evidence in a recent review,^24^ but we did not find any evidence in the included studies to quantify these measures. Perioperative pregabalin administration reduced the incidence of chronic neuropathic pain in 1 study^17^ but has been refuted in a meta-analysis.^63^ Continuous regional anesthesia provided analgesic benefit for up to 1 month after surgery but did not influence PPSP outcomes at 6 months,^15^ and single-injection femoral nerve block did not affect PPSP.^128^ Because these findings are based on a limited number of studies, it is impossible to draw robust conclusions on the effectiveness of these perioperative interventions to prevent PPSP.

### 4.2. Hip joint replacement surgeries

The results of the meta-analyses in hip joint replacement surgeries only from 5 studies could not demonstrate any statistically significant results for factors consistently associated with an increased risk of PPSP. The qualitative summary from multivariable analysis results from 10 studies pointed out several possible factors associated with PPSP; however, there is insufficient evidence to conclude these contributing factors because of a small number of studies reporting them. Young age,^91^ female sex,^103^ higher time spent with pain in the perioperative period, presurgical anxiety or depression, higher BMI, poor Harris Hip Score,^11^ presence of loose beads from the prosthesis, pain at other sites of the body, preoperative pain disability, higher cumulative opioid consumption,^52^ and high intraoperative nociception were the factors reported to be associated with an increased risk of PPSP in some, but not other studies. The current data on preoperative education of patients undergoing THR are unclear,^32,46,68^ and we did not find any conclusive evidence on PPSP prevention with this approach. Perioperative ketamine was associated with a reduction in the risk of PPSP in 1 study^87^ but has been refuted in a meta-analysis.^49^ A single perioperative dose of gabapentin,^21^ spinal ketorolac, local infiltration analgesia, and femoral nerve block did not show a protective effect on the presence of PPSP at 6 months after THR. These findings are based on limited data; therefore, no conclusion can be drawn of effective strategies for preventing PPSP after THR.

### 4.3. Hip and knee joint replacement surgeries

The results of the meta-analyses in studies that combined patients undergoing hip and knee joint replacement surgeries demonstrated an increased risk of PPSP in women and patients with higher BMI, but the number of studies is likely too small to draw any meaningful conclusions. The qualitative summary of multivariable analyses from 11 studies pointed out several possible factors associated with PPSP. Functional severity of joint pathology, higher time spent with pain in the preoperative period, presurgical anxiety or depression, and presence of pain in other areas of the body were associated with higher instances of PPSP, whereas preoperative analgesia and neuraxial anesthesia were associated with less PPSP in 1 study. It has been reported that younger patients are more likely to develop PPSP after orthopedic surgery,^8^ but this finding was not consistent across studies.^75^ Mechanisms explaining the increased incidence of chronic pain in younger patients are unknown but may relate to a reduction in peripheral nociceptive function and density with increased age.^129^ It has been reported that women experience higher pain intensity at a lower level of inflammation after knee surgery compared with men, which has been attributed to central mechanisms of pain perception.^101^ Data from the American College of Surgeons National Surgical Quality Improvement Program database has shown that the degree of obesity (high BMI) relates to the of risk of postoperative complications in hip and knee arthroplasty,^92^ which is consistent with our findings from the qualitative summary. Several factors, such as employment status, illiteracy, history of hypertension or diabetes, and previous knee or hip replacement surgeries have been reported to be associated with higher PPSP in the literature but did not show association in this combined analysis.^83,84^

### 4.4. Assessment of chronic pain

Chronic pain as a subjective lived, and sensory experience presents particular challenges to assessment.^7^ This review has identified the following tools used for assessing chronic pain: Oxford Knee Score (joint-specific questionnaire assessing pain severity and interference), Western Ontario and McMaster Universities Osteoarthritis Index, Knee Injury and Osteoarthritis Outcome Score (disease-specific questionnaire assessing pain severity), the Brief Pain Inventory (disease-agnostic questionnaire with a pain severity subscale [4 items] and a pain interference subscale [7 items]), Short-Form McGill Pain questionnaire (disease-agnostic questionnaire assessing sensory and affective qualities of pain), Pain visual analogue scale (unidimensional assessment of pain severity), PainDETECT (a neuropathic pain questionnaire), and Douleur Neuropathique 4 (DN4, a neuropathic pain questionnaire). Because these tools are selected based on study objectives and each measure a somewhat different dimension of pain severity, characteristics, and interference, direct comparison among studies is challenging. It is increasingly recognized that the accurate assessment of patient-reported outcome measures (PROMs) in orthopedic surgery trials and registries is important and ideally should include metrics of quality of life, functioning, disability, and patient satisfaction.^9,66^ In both clinical and research settings, the approach to assessing chronic pain after THR or TKR needs to be in-depth and multidimensional to understand the characteristics and impact of this pain. Unfortunately, the heterogeneity in PROM assessment in the included studies did not allow to robustly test the associations between PPSP and PROMs. We could not identify risk factors that are associated with diminished pain-related quality of life after knee and hip joint replacement surgeries. Only 1 study showed diminished quality of life in patients with PPSP compared with those without,^36^ while 2 other studies focused on quality of life measures in the context of perioperative anesthesia selection^127^ and general longitudinal outcomes of joint replacement surgery.^69^

### 4.5. Quantitative sensory testing modalities

Quantitative sensory testing (QST) is a psychophysical approach used to quantify somatosensory function in response to controlled stimuli.^5^ The idea of using QST in the preoperative setting to determine an individual's response to painful stimuli is appealing as a possible predictor of a response to a painful surgical procedure. In this review, heat pain threshold such as hyperalgesia was associated with perioperative pain after TKR^62^ and preoperatively facilitated TSP and CPM assessed using mechanical stimuli or manual cuff stimuli with knee osteoarthritis.^78,80,125^ Hypoalgesia after cold pressor stimulation and aerobic exercise assessed preoperatively by cuff algometry was associated with pain relief 6 months after TKR.^110^

### 4.6. Potential modifiers

Certain factors such as anxiety, depression, catastrophizing, and BMI are potentially modifiable, and future studies should investigate whether preoperative modification of these factors can mitigate PPSP risk. Cognitive behavior therapy and other holistic approaches may be relevant in this setting.^118^ Other risk factors such as age, and sex are not modifiable; however, they may be important to developing risk stratification tools. Intraoperative and perioperative factors such as surgical and anesthesia techniques as well as perioperative care and analgesia plans can be modified to mitigate the risk for PPSP in these patients.

### 4.7. Limitations of our study

It is important to acknowledge the limitations of this review when interpreting the results. First, although we performed a systematic literature search, gray literature as well as abstracts and dissertations were not evaluated, potentially missing some studies. Some full-text articles described data in a format which could not be retrieved for meta-analyses; these data were only included in a qualitative summary. We contacted authors in such situations but were only successful to obtain additional data in a few cases. We included a mix of both retrospective and prospective studies, as well as observational and interventional studies. There are no guidelines how to weigh risk factors based on differences in study design,^23^ and this may have led to heterogeneity in the study population and recall bias in retrospective studies.^41^ Second, with the main outcome of PPSP occurrence as a categorical variable, it is also possible that we missed certain constructs of PPSP as captured by multidimensional or descriptive tools, such as PROMs^66^ after hip and knee arthroplasty which has been well described elsewhere.^40,44,61,117,124,130^ Third, this review included data both from studies on THR and TKR, although the literature has shown that pain and patient satisfaction outcomes from THR are generally better in comparison with TKR.^64^ However, we analyzed the data separately, where possible. Fourth, we could not account for the effect of pharmacological and nonpharmacological interventions on the reporting of pain from the available data. For example, there were several predictor variables reported as important in the individual studies, but for which not enough data were available to conduct a meaningful meta-analysis. These include coping skills, negative beliefs about opioids having a higher PPSP risk,^22^ biomechanical factors, and QST measures. These factors were qualitatively summarized based on multivariate analyses in some studies but require further and more rigorous evaluation, ideally in the setting of multicenter studies. Several of the included studies described perioperative interventions that were available only from 1 study.^14,18,115,128,19,21,36,51,76,86,88,96^ We did not exclude them and summarized their findings qualitatively; however, the ability to draw generalizable conclusions from these studies remains limited. Fifth, there was also marked heterogeneity in the study designs, interventions (eg, spinal anesthesia may offer shorter duration of analgesia compared with continuous epidural analgesia^31^), statistical analyses undertaken, and ROB scores. THA or TKA may be performed under general, regional, or neuraxial anesthesia (or a combination of these); the choice may be based on institutional practices, patient comorbidities, and patient preference.^28^ In this review, 4 articles focused on regional anesthesia^14,51,76,128^ and 2 on intrathecal analgesia.^96,115^; however, with insufficient information to draw conclusions on differences in anesthetic approaches in the context of PPSP prevention. Finally, the follow-up periods were grouped to a mean of 6 months postoperatively (as per inclusion criteria) so some precision may be lost in the meta-analyses. As no widely available risk of the bias assessment system is available for a group of studies with mixed designs, we used previously published tools to adapt to the needs of this review; it has been used in reviews with similar methodology^56^; however, the entire battery of questions has not been validated as a single tool.

## 5. Conclusions

Our review systematically addressed factors associated with risk for PPSP and identified that higher preoperative state anxiety and depression increase the risk of PPSP after TKR. No consistent risk factors for PPSP after THR were identified in the meta-analysis. In the qualitative summary of multivariable analyses performed in individual studies, higher preoperative pain scores and more severe acute postoperative pain were associated with a higher risk of PPSP after TKR or THR. There was a wide variation in the methods of pain assessment and in the tools used to collect perioperative variables. Standardization of tools and methods for perioperative data collection and PPSP assessment, improvement in follow-up rates, proper characterization of patients who dropped off from the studies, and ascertaining the reproducibility of PPSP prevention strategies by conducting multicenter studies can all improve the understanding and mitigation strategies of PPSP after joint replacement surgeries.

## Disclosures

S. Haroutounian reports research grants from Disarm Therapeutics and personal fees from Rafa Laboratories and Vertex Pharmaceuticals, outside the scope of this paper. The remaining authors have no conflicts of interest to declare.

## Appendix A. Supplemental digital content

Supplemental digital content associated with this article can be found online at http://links.lww.com/PR9/A184.
